# P-1491. Invasive Meningococcal Disease Incidence and Risk Among Commercially- and Medicaid-Insured Infants in the United States

**DOI:** 10.1093/ofid/ofaf695.1675

**Published:** 2026-01-11

**Authors:** Oscar Herrera-Restrepo, Elizabeth Packnett, Megan Richards, Elise Kuylen, Tosin Olaiya, Thatiana Pinto, Lindsay Landgrave, Andrew G Allmon

**Affiliations:** GSK, Philadelphia, PA; Merative, Ann Arbor, Michigan; Merative, Ann Arbor, Michigan; GSK, Philadelphia, PA; GSK, Philadelphia, PA; GSK, Philadelphia, PA; GSK, Philadelphia, PA; GSK, Philadelphia, PA

## Abstract

**Background:**

Invasive meningococcal disease (IMD), caused by *Neisseria meningitidis*, is an uncommon but serious disease. The highest incidence rates (IRs) in the United States (US) are reported among infants (< 1 year). In this study, we estimated IMD IRs by patient characteristics (month [m] of age and clinical conditions) among commercially- and Medicaid-insured US infants.

**Figure:**
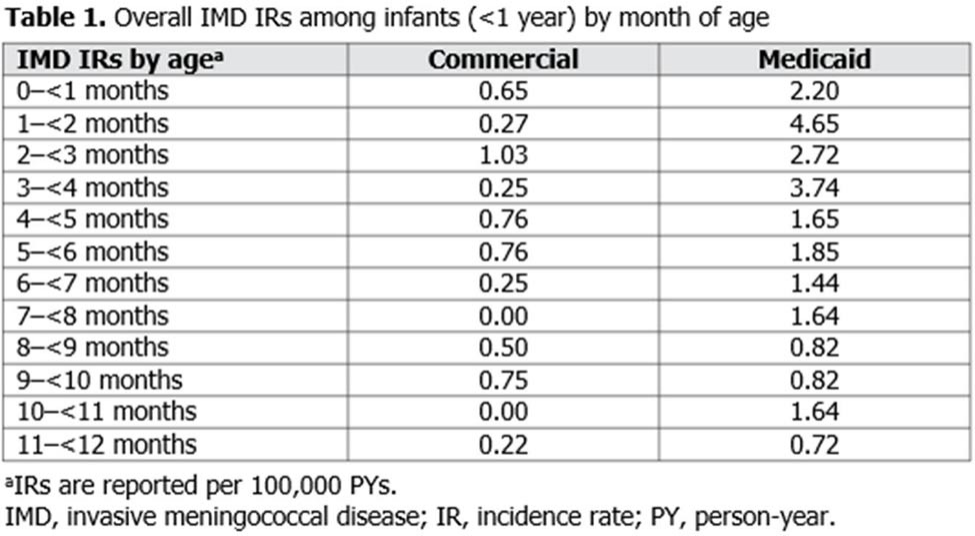

**Figure:**
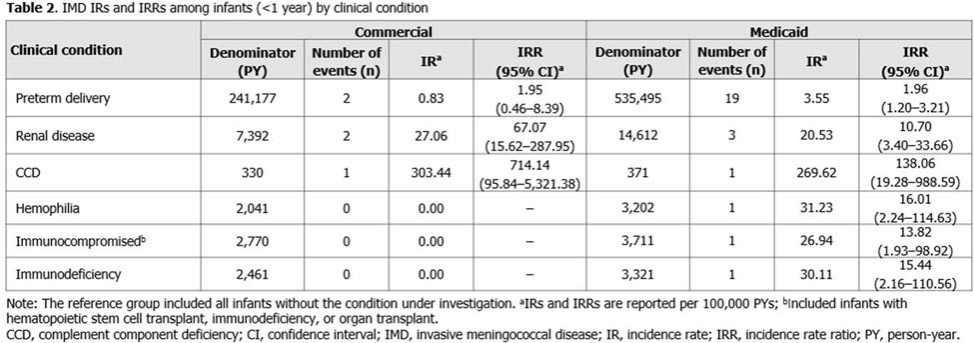

**Methods:**

Claims data of commercially- and Medicaid-insured infants from 01/2005–12/2022 were analyzed. Eligibility was based on birth dates (index date: first date patient met the age and enrollment criteria). Conditions known to increase IMD risk (eculizumab use, asplenia, human immunodeficiency virus, complement component deficiency [CCD], sickle cell disease) and those hypothesized to increase IMD risk (asthma, autoimmune disease, type 1 diabetes, corticosteroid use, immunocompromised status, malignancy, preterm delivery, renal disease) were assessed. IRs were estimated per 100,000 person-years (PYs); IR ratios (IRRs) and 95% confidence intervals were calculated to compare infants with each clinical condition to those without the condition.

**Results:**

In the commercial (N=7.2 million) and Medicaid (N=7.4 million) cohorts, 21 and 115 infants were diagnosed with IMD, respectively. The overall IMD IR was higher in the Medicaid cohort (IR=1.97) versus the commercial cohort (IR=0.45). In the Medicaid cohort, the highest IRs were observed for infants aged 0–< 1 m (IR=2.20), 1–< 2 m (IR=4.65), 2–< 3 m (IR=2.72), and 3–< 4 m (IR=3.74). The highest IR in the commercial cohort was observed for infants aged 2–< 3 m (IR=1.03; Table 1). IRRs across both cohorts were >1 among infants with preterm delivery (commercial: n=2, IR=0.83; Medicaid: n=19, IR=3.55), renal disease (commercial: n=2, IR=27.06; Medicaid: n=3, IR=20.53), and CCD (commercial: n=1, IR=303.44; Medicaid: n=1, IR=269.62; Table 2).

**Conclusion:**

Overall, IMD incidence was low. IRs were higher among Medicaid-insured infants, infants aged < 4 m, and infants with certain clinical conditions. Whilst targeted mitigation strategies may reduce IMD burden among high-risk groups, difficulty in identifying individuals at increased risk, especially at an early age, may necessitate a broader approach.

Funding: GSK VEO-000994

**Disclosures:**

Oscar Herrera-Restrepo, PhD, GSK: Employee|GSK: Stocks/Bonds (Public Company) Elizabeth Packnett, MPH, GSK: Grant/Research Support|Merative: Employee Megan Richards, PhD, MPH, GSK: Grant/Research Support|Merative: Employee Elise Kuylen, PhD, GSK: Employee|GSK: Stocks/Bonds (Public Company) Tosin Olaiya, MBChB, MSc, GSK: Employee|GSK: Stocks/Bonds (Public Company) Thatiana Pinto, PhD, GSK: employee|GSK: Stocks/Bonds (Public Company) Lindsay Landgrave, PharmD, GSK: Employee|GSK: Stocks/Bonds (Public Company) Andrew G. Allmon, DrPH, GSK: Employee|GSK: Stocks/Bonds (Public Company)

